# Deciphering the bacterial composition in the rhizosphere of *Baphicacanthus cusia* (NeeS) Bremek

**DOI:** 10.1038/s41598-018-34177-1

**Published:** 2018-10-25

**Authors:** Meijuan Zeng, Yongjia Zhong, Shijie Cai, Yong Diao

**Affiliations:** 1 School of Biomedical Sciences, Huaqiao University, 362021 Quanzhou, China; 2 Zhangzhou Health Vocational College, 363000 Zhangzhou, China; 3 Root Biology Center, Fujian Agriculture and Forestry University, 350002 Fuzhou, China; 4 Institute of Genetics and Developmental Biology, Chinese Academy of Sciences, 100101 Beijing, China; 5 Nuffield Division of Clinical Laboratory Sciences, University of Oxford, John Radcliffe Hospital, Headington, Oxford, OX3 9DS UK

## Abstract

Rhizobacteria is an important ingredient for growth and health of medicinal herbs, and synthesis of pharmacological effective substances from it. In this study, we investigated the community structure and composition of rhizobacteria in *Baphicacanthus cusia* (NeeS) Bremek via 16S rRNA amplicon sequencing. We obtained an average of 3,371 and 3,730 OTUs for bulk soil and rhizosphere soil samples respectively. Beta diversity analysis suggested that the bacterial community in the rhizosphere was distinctive from that in the bulk soil, which indicates that *B.cusia* can specifically recruit microbes from bulk soil and host in the rhizosphere. *Burkholderia* was significantly enriched in the rhizosphere. *Burkholderia* is a potentially beneficial bacteria that has been reported to play a major role in the synthesis of indigo, which was a major effective substances in *B. cusia*. In addition, we found that *Bacilli* were depleted in the rhizosphere, which are useful for biocontrol of soil-borne diseases, and this may explain the continuous cropping obstacles in *B. cusia*. Our results revealed the structure and composition of bacterial diversity in *B. cusia* rhizosphere, and provided clues for improving the medicinal value of *B. cusia* in the future.

## Introduction

Plant roots grow into the soil and are continuously in contact with the microbes living in the soil. Rhizosphere is a narrow interface between plant roots and soils for energy and material exchange. Rhizosphere microbes are affected by root exudation. This area contains up to 10^11^ microbial cells per gram root^1^. The microbes living in this narrow play a vital role in plant growth and health. Microbes help to increase the bioavailability of important mineral nutrients such as N, P and K^2^. Another beneficial function of rhizobacteria is to suppress soil-borne diseases^3,4^. In terms of medicinal herb research, the rhizosphere bacteria also influence the synthesis of effective substances^5^. The plants in turn feed the microbes in the rhizosphere with carbohydrates derived from photosynthesis in the form of rhizodeposition^6^. It has been reported that about 17% of photoassimilates are released into the rhizosphere in the form of rhizodeposition^7^, which results in the recruitment and enrichment of beneficial or detrimental soil bacteria from bulk soil^8^. Hence, the microbes living in the rhizosphere of the plant can be divided into beneficial microbes, neutral microbes and detrimental microbes. The neutral microbes are harmless to plants. Beneficial microbes can dissolve some insoluble minerals, and promote plant growth or provide phytohormones such as IAA, while the detrimental microbes can cause plant diseases by producing toxic substances. The rhizosphere bacteria are dominated by bacteria, fungi, actinomycetes, algae, protozoa, etc^9^. Bacteria are the most abundant microorganisms in the soil^10^. The analysis of abundance of microbes in the rhizosphere of Paris polyphylla var. yunnanensis showed the following relationship^11^: bacteria > actinomycetes > fungi. The classification and identification of bacteria are developed from phenotypic characteristics identification to genetic characteristics classification. In addition, the composition of rhizosphere community is determined by the soil type and plant genotype^12^. Hence, understanding the composition of bacterial community in the nature is important for the utilization of beneficial bacteria to improve the production and quality of medicinal herbs.

The rhizosphere is one of the most complex ecological niches in the nature^13^, which makes it difficult to investigate the composition and function of the bacteria in it. High-throughput sequencing has facilitated major advances in the understanding of microbial ecology. The 16S rRNA gene of bacteria and archaea are frequently used to characterize the taxonomic composition, phylogenetic diversity and microbial community composition^14^. 16S rRNA is located in prokaryotic small subunit ribosome, and includes ten conserved regions and nine hypervariable regions^15^. This technology has been used to investigated the microbial composition in different plants, such as *Arabidopsis* accessions^16–18^, maize^19^, *Populus deltoids*^20,21^ and rice^22^. However, there are very few reports on the bacterial community in medicinal herb roots or rhizosphere.

*Baphicacanthus cusia* (Nees) Bremek (Figure S1) is a common medicinal herb in China, which is usually used in Traditional Chinese Medicine (TCM). Its underground roots are often used as raw materials to produce radix isatidis that has been listed in the Chinese Pharmacopoeia^23^. As an important medicinal herb, it is widely cultivated in Southern and Eastern China^24,25^. *B. cusia*, with its antibacterial and antiviral properties^26^, is often used to treat colds, fever, meningitis, and other symptoms^27^. Its leaves and stems are important source of Qing Dai, which is useful to treat diseases such as ulcerative colitis^28^, leukemia^29^, and psoriasis^30^. Indigo, indirubin and tryptanthrin are reported to be the major effective substances of *B. cusia* responsible for its anti-inflammatory and anti-tumor effects^31–36^. Tryptanthrin can inhibit multi-drug resistance gene expression, and exhibits anti-inflammatory effect by inhibiting nitric oxide (NO) synthesis^37^. However, due to continuous cropping obstacle, *B. cusia* has to be transplanted after every three years or else has the risk of poor growth^38–40^. Many plants have various degrees of continuous cropping obstacles. Related researches have focused on the cause of continuous cropping obstacle in the deterioration of physicochemical properties, microbial community structure and diversity imbalances and the changes in enzyme activity of continuous cropping soil^41^. The problem of continuous cropping obstacle is very common in medicinal plants, especially in rhizomatous medicinal plants, such as *Pseudostellaria heterophylla(Miq)Pax*., *Angelica sinensis (Oliv.) Diels*., *Ligulariaduciformis (C.Winkl.) Hand.-Mazz*., *Coptischinensis Franch*. and *Panaxginseng C. A. Mey*.^42^. *B. cusia*, it is less likely to be attacked by pathogens and pests, but is susceptible to root rot^43^. Root rot is closely related to rhizomatic pathogenic bacteria^44^, which is usually caused by breaking the homeostasis of rhizobacterial community. Therefore, revealing the bacterial community composition of *B. cusia* is essential for understanding the underlying mechanism. In this study, we investigated the structure and composition of *B. cusia* rhizosphere soil and bulk soil by employing an Illumina-based sequencing approach targeting the V4 hypervariable regions of the 16S rRNA gene. This research provided the theoretical basis for exploring the relationship between *B. cusia* and rhizobacteria, which can uncover the relationship between continuous cropping obstacle and rhizobacteria in *B. cusia*. All these can lead to finding a new way to improve the yield and quality of *B. cusia*.

## Results

### Overall analysis of bacterial community in bulk soil and *B. cusia* rhizosphere

**Figure 1:**
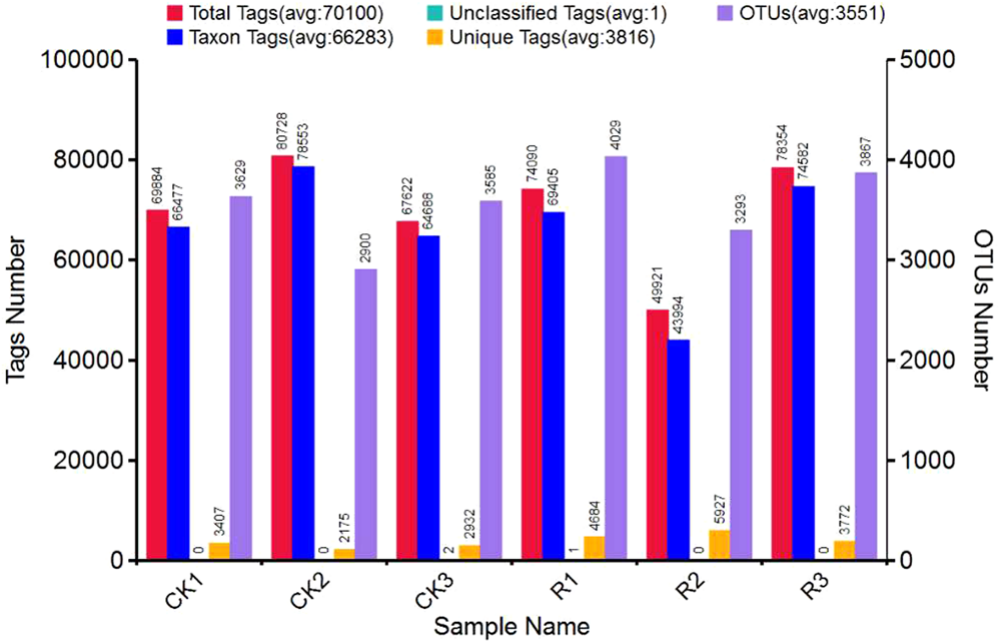
Operational Taxonomic Units (OTUs) analysis of *B.cusia* rhizosphere soil and bulk soil. The horizontal axis presents the sample name, the first vertical axis presents tags number, and the second vertical axis presents the OTUs number. R: rhizosphere soil, CK: bulk soil.

**Figure 2:**
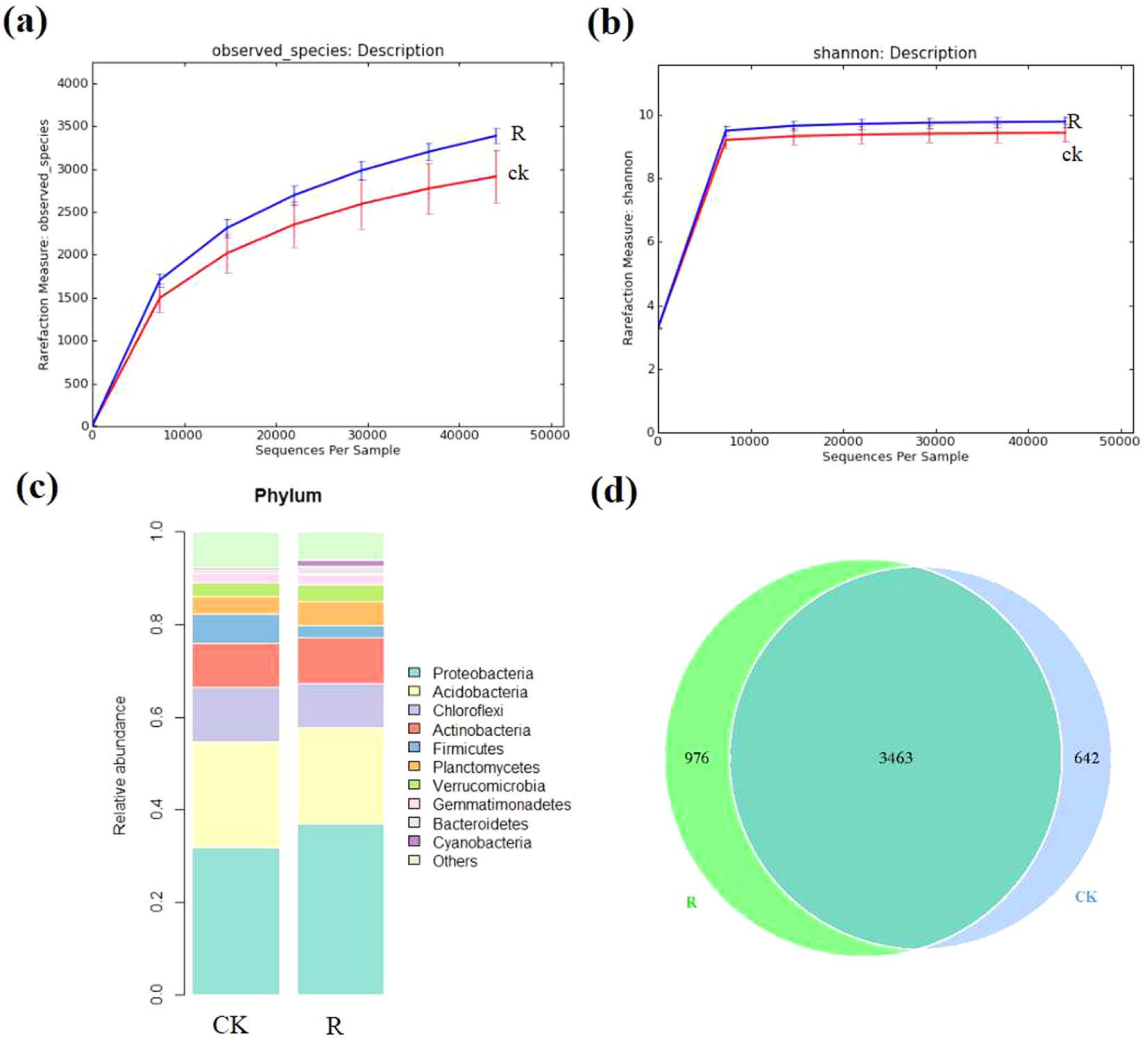
Overall analysis of bacterial communities in *B. cusia* rhizosphere soil and bulk soil. (**a**) Rarefaction curve of observed species between *B. cusia* rhizosphere soil and bulk soil. (**b**) Rarefaction curve of Shannon index between *B.cusia* rhizosphere soil and bulk soil. (**c**) The bacterial composition of *B. cusia* rhizosphere soil and bulk soil at the phylum taxonomic level. (**d**) The Venn map of bacterial communities in *B. cusia* rhizosphere soil and bulk soil. There were 3463 OTUs both shown in bulk soil and *B.cusia* rhizosphere, and 642 OTUs were only in bulk soil and 976 OTUs were only shown in the rhizosphere samples. R: rhizosphere soil, CK: bulk soil.

Through 16S rRNA sequencing of all bulk soil and rhizosphere soil samples, we obtained a total of 420,599 total tags. After quality control, a total of 397,699 taxon tags were obtained. We picked the operational taxonomic units (OTUs) to create an OTUs table. Sequences with 97% similarity were assigned to the same OTU. We obtained an average of 3,371 OTUs for bulk soil and 3,730 OTUs for rhizosphere soil samples (Fig. 1). The rarefaction curve of observed species showed that the sequencing depth was sufficient to cover detectable species in both bulk soil and rhizosphere soil samples, since the curve had almost plateaued (Fig. 2a). In addition, the rarefaction curve of Shannon index was consistent with the observed species (Fig. 2b). We also analyzed the bacterial composition at the phylum taxonomic level, which showed that *B. cusia* rhizosphere and bulk soils were dominated by *Proteobacteria*, *Acidobacteria*, *Chloroflexi*, *Actinobacteria*, *Firmicutes*, *Planctomycetes*, *Verrucomicrobia*, *Gemmatimonadetes*, *Bacteroidetes* and *Cyanobacteria* (Fig. 2c). The Venn map showed that 3463 OTUs existed in bulk soil and *B. cusia* rhizosphere, while 642 OTUs were only enriched in bulk soil and 976 OTUs in rhizosphere (Fig. 2d). As shown in Table 1, these indices showed that the diversities of bacterial communities in rhizosphere soil were higher than in bulk soil. Among the Observed species, ACE, Chao1 index, Shannon index and Simpson’s index, only ACE showed significant difference between the rhizosphere soil and bulk soil, but no significant difference were found in other indices.

**Table 1:** Bacterial diversity index in rhizosphere soil and bulk soil of *B. cusia*.

Index	Rhizosphere (n = 3)	Bulk soil (n = 3)	P-value
Observed species	3387 ± 61.67	2912 ± 215.7	0.1507
ACE	4388 ± 156.4	3547 ± 200.8	0.0325
Chao1 index	5211 ± 950.6	3452 ± 211.9	0.2013
Shannon index	9.783 ± 0.113	9.434 ± 0.1976	0.2174
Simpson’s index	0.9967 ± 0.0003	0.9963 ± 0.0007	0.6856

Notes: P-value indicated were significant difference between R and CK using *t*-test.

### Bacterial diversity in *B. cusia* rhizosphere

**Figure 3:**
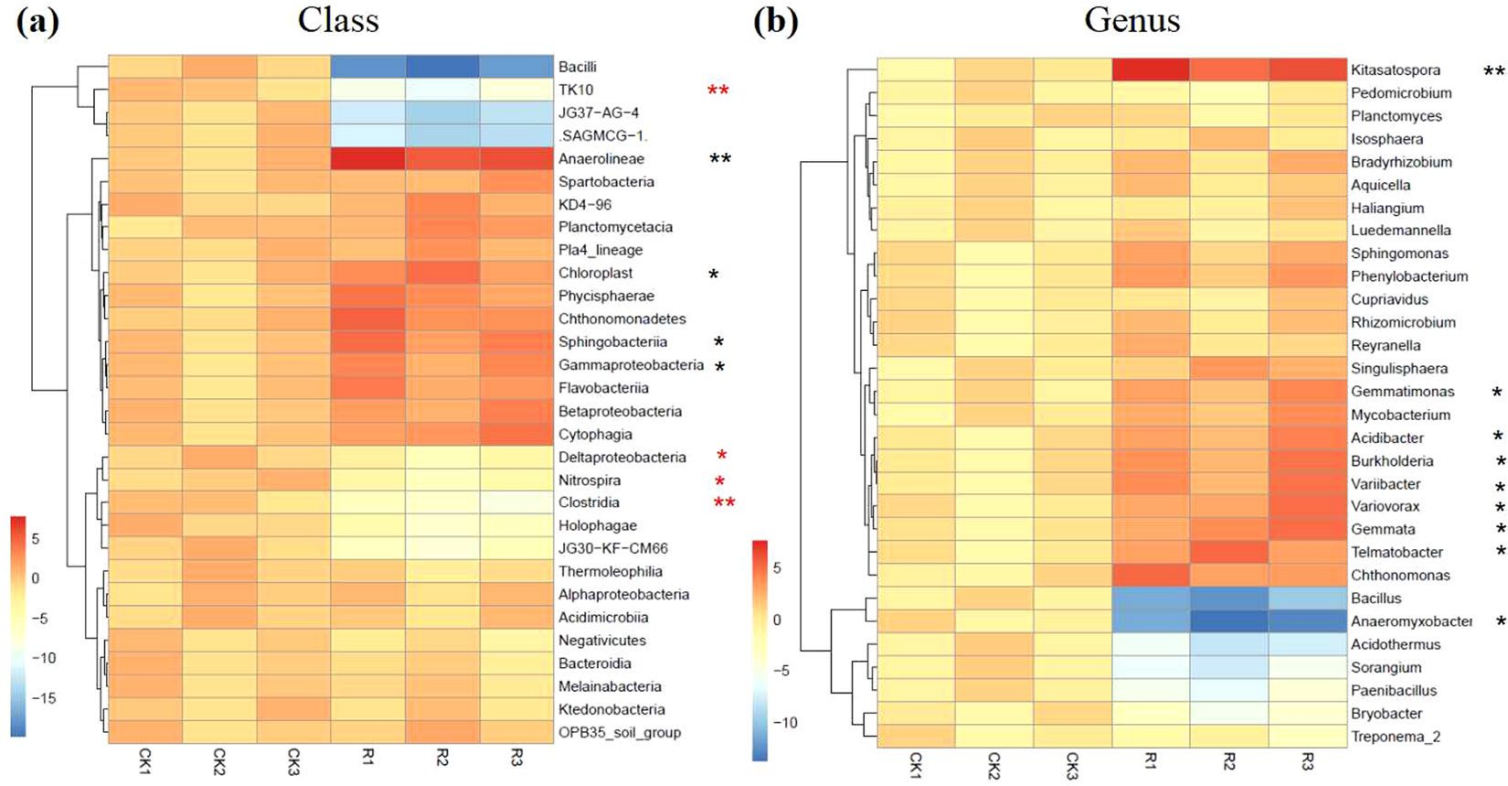
The top 30 relative abundance of bacteria in *B. cusia* rhizosphere soil and bulk soil at different taxonomic level. (**a**) The top 30 relative abundance of bacteria at the class taxonomic level. (**b**) The top 30 relative abundance of bacteria at the genus taxonomic level. R: rhizosphere soil, CK: bulk soil (**p < 0.01; *p < 0.05).

To analyze the bacterial structure and composition, we examined the bacterial relative abundance in rhizosphere soil at different taxonomic levels. We mainly presented the top 30 relative abundance of bacteria. At the class taxonomic level, the top five bacteria with relative high abundance were: *Alphaproteobacteria, Betaproteobacteria, Deltaproteobacteria, Ktedonobacteria* and *Gammaproteobacteria. Anerolineae*, *Chloroplast, Sphingobacteriia* and *Gammaproteobacteria* were significantly enriched in rhizosphere soil. In contrast, the *TK10*, *Deltaproteobacteria*, *Nitrospira* and *Clostridia* were significantly depleted in the rhizosphere as compared to the bulk soil. *Bacilli* were also depleted in the rhizosphere as compared to the bulk soil, but was not significant (Fig. 3a). At the genus taxonomic level, the top five bacteria with relative high abundance were *Acidothermus, Acidibacter, Bacillus, Bradyrhizobium and Bryobacter*. In addition, *Kitasatospora*, *Anaeromyxobacter*, *Gemmatimonas*, *Acidibacter*, *Burkholderia*, *Variibacter*, *Variovorax*, *Gemmata* and *Telmatobacter* were significantly enriched in the rhizosphere. *Bacilus* was also depleted in the rhizosphere as compared to the bulk soil, but the difference was not significant (Fig. 3b).

### *B.cusia* recruits special microbes from bulk soil and hosts a distinctive bacterial community in the rhizosphere

**Figure 4:**
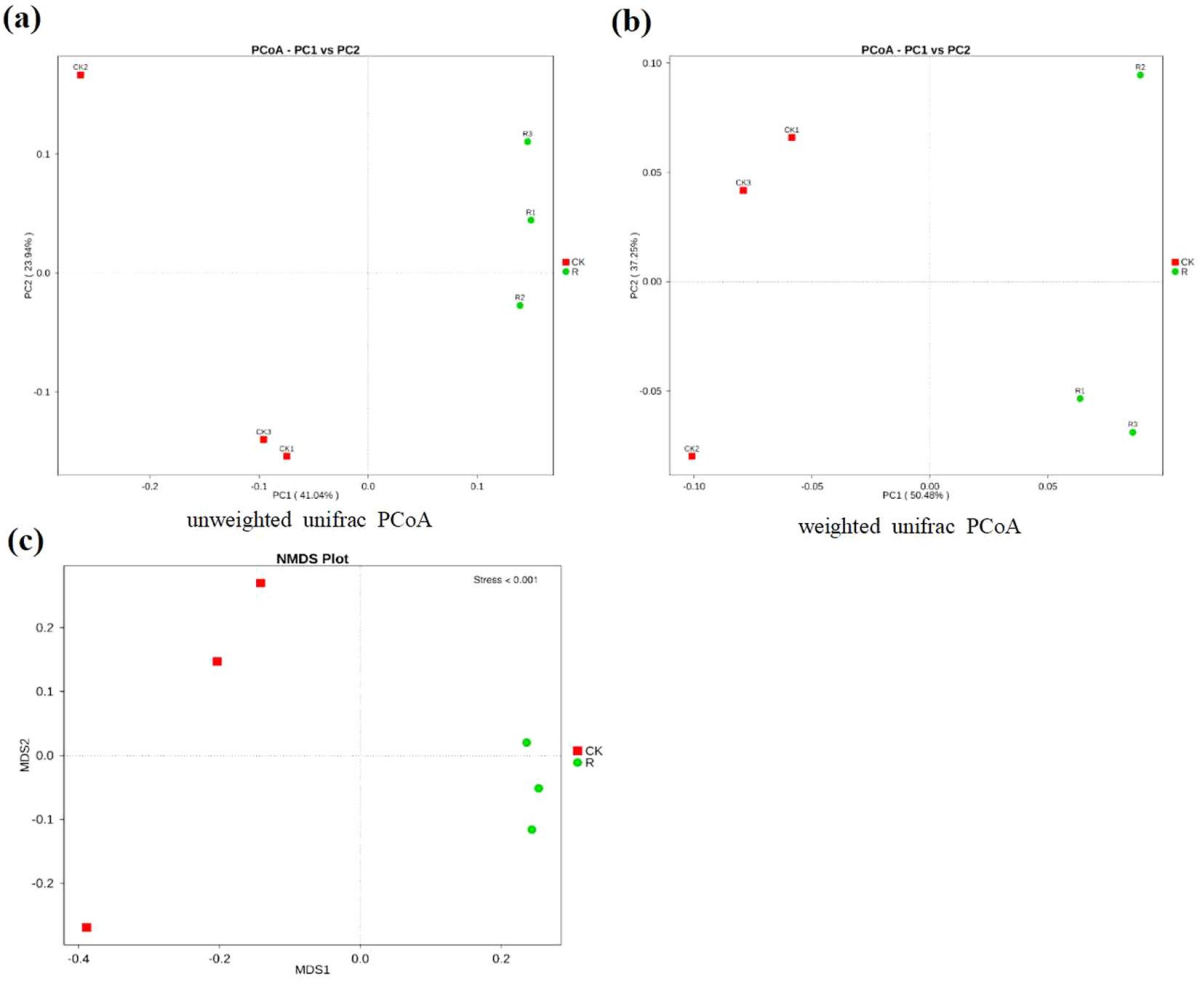
Principal Coordinates Analysis (PCoA) and Nonmetric Multidimensional Scaling (NMDS) of bacterial communities in *B. cusia* rhizosphere soil and bulk soil. (**a**) Unweighted unifrac PCoA of bacterial communities in *B.cusia* rhizosphere soil and bulk soil. PC1 explained 41.04% of the variation while PC2 explained 23.94%. (**b**) Weighted unifrac PCoA of bacterial communities in *B. cusia* rhizosphere soil and bulk soil. PC1 explained 50.48% of the variation while PC2 explained 37.25%. (**c**) NMDS of bacterial communities in *B. cusia* rhizosphere soil and bulk soil. R: rhizosphere soil, CK: bulk soil.

**Figure 5:**
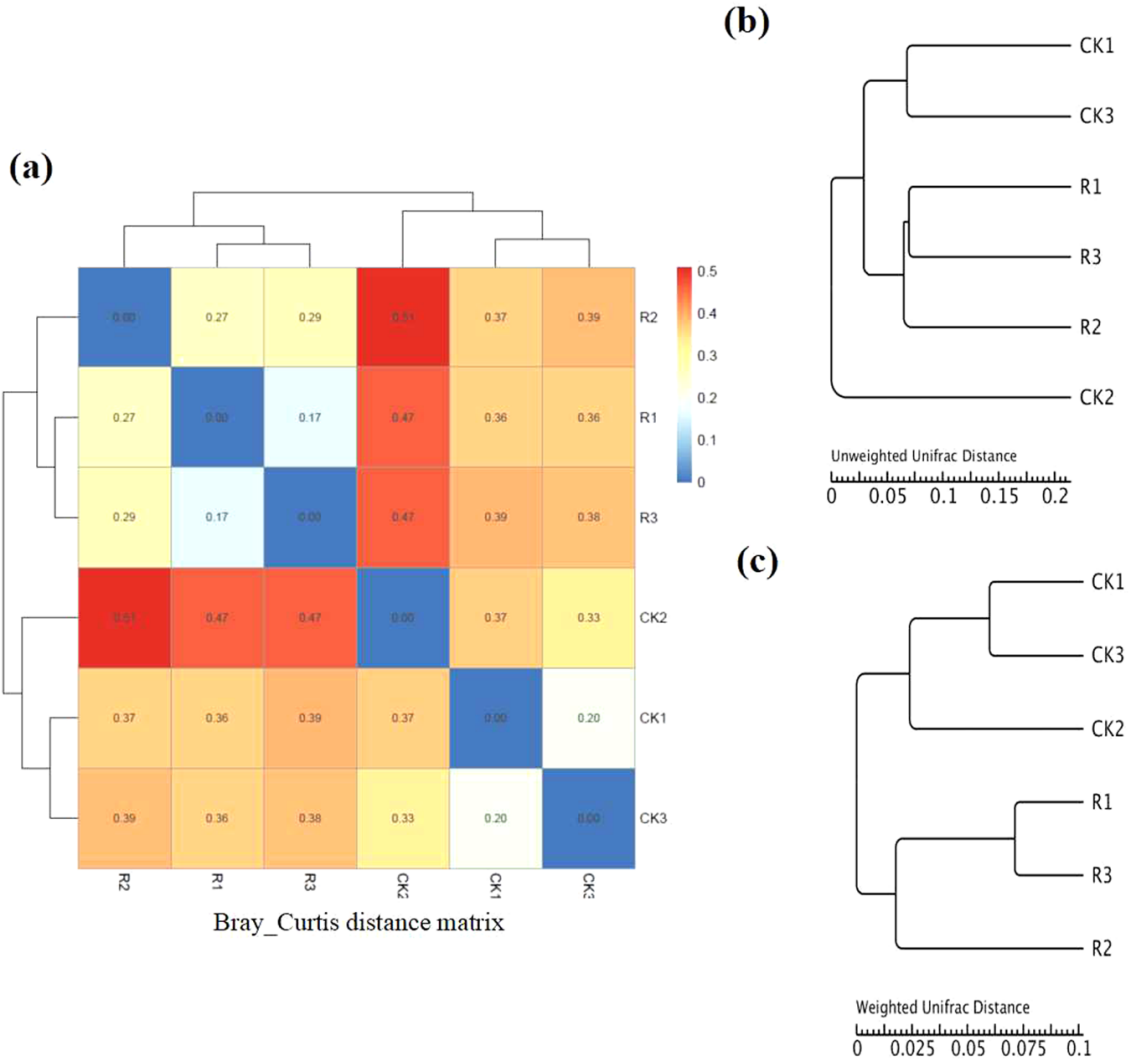
Correlation of bacterial communities between *B. cusia* rhizosphere soil and bulk soil. (**a**) Bray_Curtis distance matrix of bacterial communities in *B. cusia* rhizosphere soil and bulk soil. (**b**) UPGMA clustering analysis using unweighted unifrac distances of bacterial communities between *B. cusia* rhizosphere soil and bulk soil at the phylum taxonomic level. (**c**) UPGMA clustering analysis using weighted unifrac distances of bacterial communities between *B. cusia* rhizosphere soil and bulk soil at the phylum taxonomic level. R: rhizosphere soil, CK: bulk soil.

**Figure 6:**
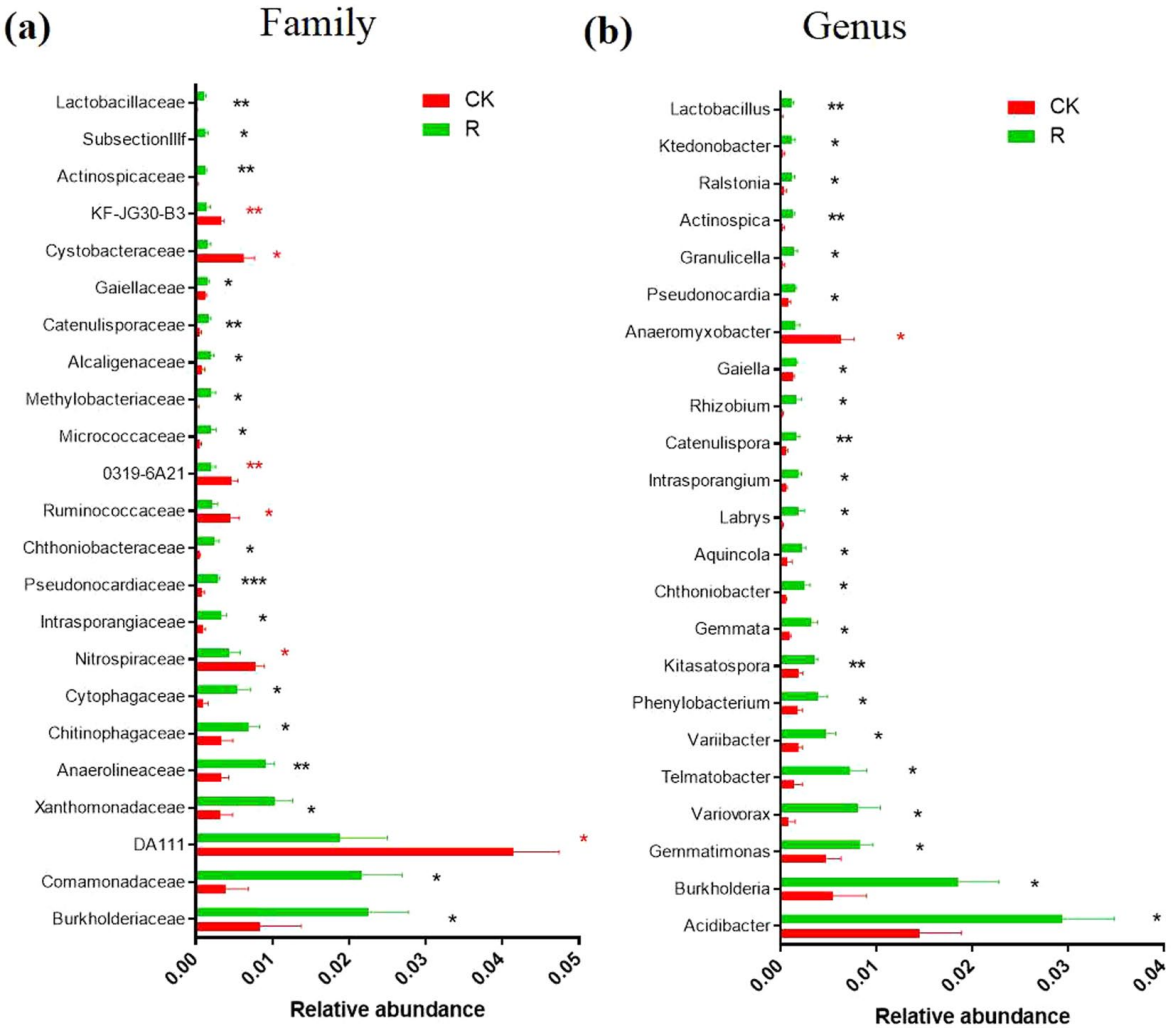
Comparison of the significant microbes between rhizosphere soil and bulk soil at different taxonomic levels. (**a**) The histogram of significant microbes between rhizosphere soil and bulk soil at the family taxonomic level. (**b**) The histogram of significant microbes between rhizosphere soil and bulk soil at the genus taxonomic level. R: rhizosphere soil, CK: bulk soil (***p < 0.001; **p < 0.01; *p < 0.05).

**Figure 7:**
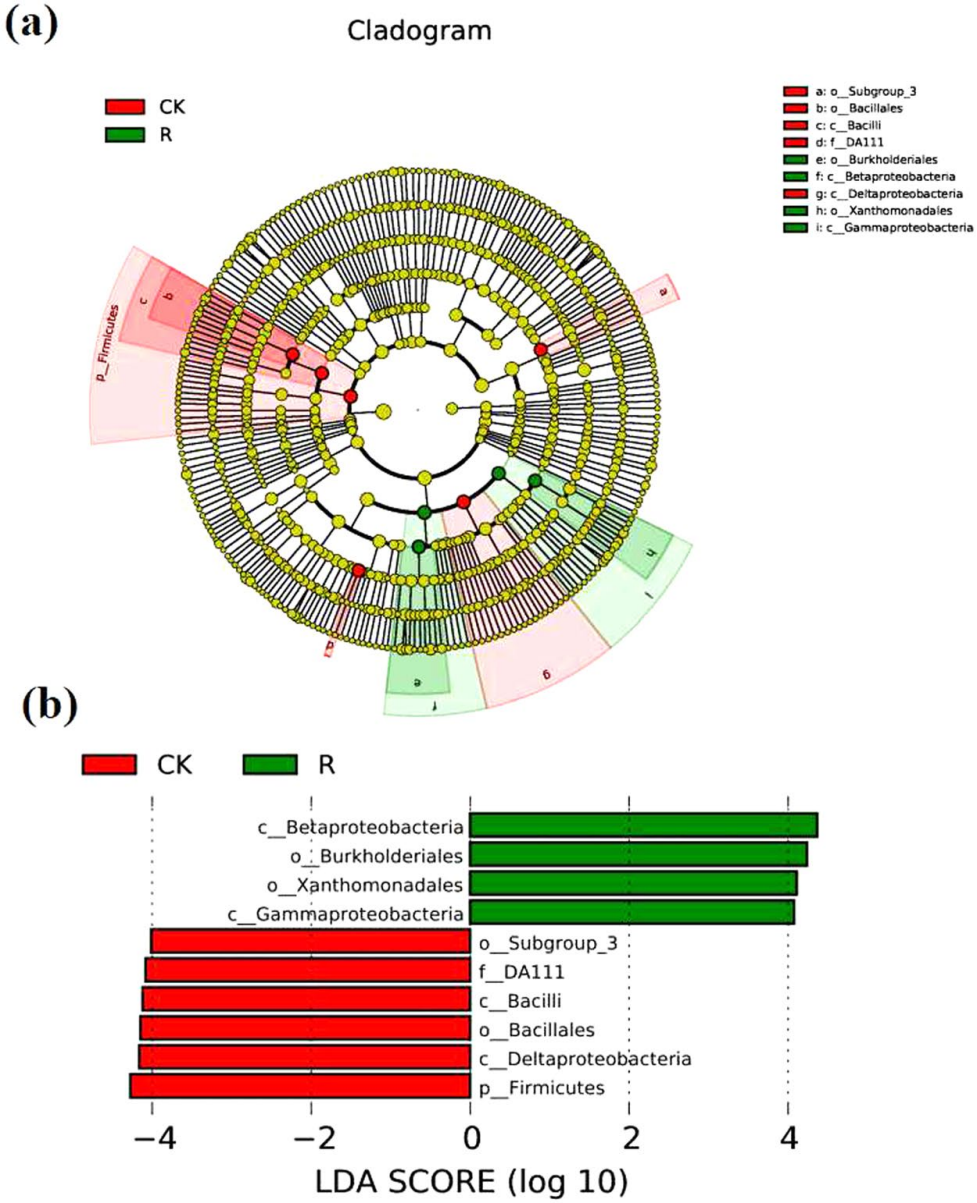
The LEfSe analysis of bacterial communities in *B. cusia* rhizosphere soil and bulk soil. (**a**) Cladogram of bacterial communities in *B. cusia* rhizosphere soil and bulk soil. (**b**) LDA scores of biomarker bacteria. LDA scores are shown as horizontal bars for the biomarker bacteria with an LDA score >4. R: rhizosphere soil, CK: bulk soil.

In order to analyze the differences in bacterial communities between rhizosphere soil and bulk soil, we performed Principal Coordinates Analysis (PCoA) and Nonmetric Multidimensional Scaling (NMDS). The results of the unweighed and weighed PCoA showed that bulk soil samples were clearly separated from rhizosphere soil samples by PC1 (unweighed PC1 = 41.04%, weighed PC1 = 50.48%). The NMDS analysis showed similar result as PCoA, which indicated that the bacterial communities in the rhizosphere were significantly different from that of bulk soil (Fig. 4). In addition, both bray_Curtis distance matrix and UPGMA clustering analysis based on weighted and unweighted unifrac distance showed that the bacterial communities in the rhizosphere were different from that of bulk soil based on their cluster pattern (Fig. 5). To further identify the microbes that were significantly enriched or depleted in rhizosphere, we analyzed the significant microbes between rhizosphere soil and bulk soil at both the family and genus levels. At the family taxonomic level, *Burkholderiaceae, Comamonadaceae, Xanthomonadaceae, Anaerolineaceae, Chitinophagaceae, Cytophagaceae, Intrasporangiaceae, Pseudonocardiaceae, Chthoniobacteraceae, Micrococcaceae, Methylobacteriaceae, Alcaligenaceae, Catenulisporaceae, Gaiellaceae, Actinospicaceae, SubsectionIIIf* and *Lactobacillaceae* were significantly enriched in the rhizosphere (Fig. 6a). At the genus taxonomic level, *Acidibacter, Burkholderia, Gemmatimonas, Variovorax, Telmatobacter, Variibacter, Phenylobacterium, Kitasatospora, Gemmata, Chthoniobacter, Aquincola, Labrys, Intrasporangium, Catenulispora, Rhizobium, Gaiella, Pseudonocardia, Granulicella, Actinospica, Ralstonia, Ktedonobacter and Lactobacillus* were significantly enriched in the rhizosphere (Fig. 6b). The LEfSe analysis showed that the biomarkers of bulk soil were *Firmicutes*, *Deltaproteobacteria*, *Bacillales*, *Bacilli*, *DA111*, and the biomarkers for *B. cusia* rhizosphere were *Betaproteobacteria, Burkholderiales, Xanthomonadales* and *Gammaproteobacteria*. The result of LEfSe analysis was consistent with the previous results indicating that *Burkholderia* was significantly enriched in the rhizosphere of *B. cusia*, while *Bacilli* exhibited low abundance in the rhizosphere as compared to the bulk soil, although not significant (Fig. 7). Taken together, these results suggested that the bacterial community in the rhizosphere of *B. cusia* was distinctive from the bulk soil and the *Burkholderia* was significantly enriched in the rhizosphere indicating that it is an important biomarker for the rhizosphere.

## Discussion

Soil has known to be one of the environments with the most diverse microbes^45^. Soil biodiversity is a key determinant of the ecological and evolutionary responses of terrestrial ecosystems to current and future environmental changes^46^. The function of soil is mainly dependent on the diversity of microbes living in it. These microbes are essential for plant nutrition and health. Rhizosphere is an important exchange interface of material and energy between plants and microbes. The rhizosphere of medicinal herbs is similar to other crops, since it is a nutrient-rich zone, where soil bacteria compete for the limited nutrients derived from plants. Furthermore, the plant-associated microbial community, which is also referred to as the second genome of the plant is crucial for plant health and growth^47^. Previous studies have shown about 75 × 10^5^ CFU of cultivable microbes per gram rhizosphere soil in burdock^48^. Zhang *et al*.^49^ also found 1549 OTUs in rhizosphere soil of Cypripedium macranthum. In this study, we obtained an average of 3,730 OTUs for *B. cusia* rhizosphere using 16S rRNA sequencing. This suggested that there were numerous bacteria in the rhizosphere of *B. cusia*, and investigation of their function requires extensive work in the future.

Taxonomic identification based molecular methods, which are independent of microbial cultivation, are widely used to investigate the composition of soil bacterial community. The basic structure of bacteria includes a cell wall that can maintain inherent shape a cell membrane that underlines the cell wall cytoplasm that plays a major role in determining its size and structural integrity^50^ and karyoplasm that controls the various bacterial genetic traits. There are three ribosomal RNAs in the bacterial cytoplasm: 16S rRNA, 23S rRNA, and 5S rRNA that they participate in bacterial protein synthesis. 16S rRNA sequence is used for microbial taxonomic identification. Recently, molecular identification methods combined with high-throughput sequencing have been widely applied in the study of bacterial community composition^51^. The application of high-throughput sequencing in the study of microbial community is based on culture independent method. For 16S rRNA amplicon sequencing, total microbial DNA was extracted to study the low abundance of microbes. To a certain extent, the relative abundance and diversity of sequence reflect relative microbial abundance and diversity in the sample^52^. Several plant microbes have been studied, including those in rice^53,54^, soybean^55^, cotton^56^ and many other plants. There are very few reports on medicinal herb microbes using high-throughput sequencing. However, there are some studies on the rhizosphere microbes of medicinal herbs. In this study, we examined a common and important medicinal herb *B. cusia* by the 16S rRNA amplicon sequencing method, and found that the diversity of bacterial community in the rhizosphere is higher than in bulk soil. However, this was only shown to be significant in the ACE index (p = 0.0325), but not in Observed species, Chao1 index, Shannon index and Simpson’s index. This result was similar to a previous study^57^ on poplar plantation which found that the bacterial community diversity of rhizosphere soil was higher than that of bulk soil, but the difference was not significant.

Plants can determine the composition of the roots, and rhizosphere microbial community secretion of root exudates can specifically stimulate or repress the microbes^58^. This phenomenon is known as the rhizosphere effect. The microbes can release soil enzymes^59^, degrade pollutants and catalyze oxidation reduction. Therefore, microbes are beneficial for nutrient cycling in soil^60,61^. Soil microbial structural stability and functional diversity played an important role in maintaining soil system health^62,63^. Rhizosphere microbes in turn exert strong effect on plant growth and development by nitrogen fixation^64,65^, phosphate solubilization^66,67^, hormone production^68,69^, and forming a plant-rhizosphere microbe interaction environment. Studies showed that roots with selectivity for rhizosphere microbes^57,70^ can attract both beneficial and detrimental microbes^8^. In this study, at the class taxonomic level, *Bacilli* were depleted in the rhizosphere as compared to the bulk soil. At the genus taxonomic level, *Burkholderia* was significantly enriched in the rhizosphere. *Bacillus* was also depleted in the rhizosphere as compared to the bulk soil. The results were similar to the LEfSe analysis. This suggests that *B. cusia* recruits special microbes from bulk soil and hosts a distinctive bacterial community in the rhizosphere. A previous study demonstrated that *Burkholderia* can present resistances to multiple heavy metals and antibiotics. It can also produce indole-3-acetic acid, 1-aminocyclopropane-1-carboxylic acid deaminase and siderophores. Inoculation with *Burkholderia* improved germination of seeds of the investigated vegetable plants in the presence of Cu, promoted elongation of roots and hypocotyledonary axes, enhanced the dry weights of the plants grown in the soils polluted with Cu and/or Pb, and increased activity of the soil urease and the rhizobacteria diversity^71^. Indigo-producing gene from *Burkholderia sp*. was cloned^72^. Indigo is the primary effective substances in *B. cusia*. Multiple *Bacillus* species are known to promote plant growth, in addition to the beneficial N^2−^ fixing activity, which can promote drought resistance in various plant models, including Arabi-dopsis^73^, Brachypodium^74^, pepper^75^ and rice^76^. *Burkholderia* may be related to effective substances in *B. cusia*, and the decrease of *Bacillus* may be related to the continuous cropping obstacle of *B. cus*ia. Further studies are needed to confirm this hypothesis. We can potentially design a management approach to control the presence of bacterial species in the soil and improve production and quality of B. cusia based on detrimental or beneficial species.

## Materials and Methods

### Sampling and Material Processing

Rhizosphere soil samples of *B. cusia* were collected from Shufeng domination Farm in Fujian, China (25°25′N 118°39′E). Sampling site: *B. cusia* field management measures were consistent. Bulk soil samples (CK) were collected from fifteen different sites away from *B. cusia* cultivation in the same field, and five sites were combined to form one biological replicate^77^. At each sampling site, soil samples were collected from five points within the 0–30 cm topsoil layer after the litter layer was removed. For rhizosphere sampling (R), *B. cusia* was dug out, and the roots with attached soils were gently shaken to remove loose soil until only firmly attached soil remained. This attached soil was collected as the rhizosphere soil using sterilized brushes. Rhizosphere soils from five strains of *B. cusia* were mixed to form one biological replicate^77^. In total, three biological bulk soil samples and three biological rhizosphere soil samples were analyzed. In addition, the rhizosphere soil samples were subjected to a more precise method for collecting rhizosphere soils through centrifugation of root washings according to Bulgarelli *et al*.^16,78^.

### DNA Extraction and PCR Amplification

Total genomic DNA from samples was extracted using CTAB method^79,80^, with minor modification. DNA concentration and purity were monitored on 1% agarose gels. V4 region of the 16S rRNA gene was amplified using 515-F (5′-GTGCCAGCMGCCGCGGTAA-3′) and 806-R (5′-GGACTACHVGGGTW TCTAAT-3′) primers^81^. PCR reactions (30 μL) included 15 μL of Phusion Master Mix (2x), 3 μL of primer (2 μM), 10 μL of gDNA (1 ng/μL), 2 μL of H_2_O. The PCR cycling program consisted of an initial denaturation step at 98 °C for 1 min, followed by 30 cycles of 98 °C for 10 s, 50 °C for 30 s, and 72 °C for 30 s, and a final 5 min extension at 72 °C.

### PCR Products Mixing and Purification

The PCR products were detected with 2% agarose gels electrophoresis^82^. PCR products with bright band between 300 and 400 bp were mixed in equal density ratios. Then, the mixture of PCR products was purified with gel extraction kit (Qiagen, Germany).

### Library Preparation and Sequencing

Sequencing libraries were generated using TruSeq® DNA PCR-free sample preparation kit (Illumina, USA) as per manufacturer’s recommendations and index codes were added. The library quality was assessed on the Qubit@ 2.0 Fluorometer (Thermo Scientific) and Agilent Bioanalyzer 2100 system. Finally, the library was sequenced on an Illumina HiSeq. 2500 platform and 250 bp paired-end reads were generated (completed by Beijing Novogene Science and Technology Co., Ltd)

### Data Analysis

Paired-end reads were merged using FLASH^83^. Quality filtering on the raw tags were performed under specific filtering conditions to obtain the high-quality clean tags^84^ according to the QIIME^85^ (http://qiime.org/index.html) quality controlled process. The tags were compared with the reference database using UCHIME algorithm^86^ (http://www.drive5.com/usearch/manual/uchime_algo.html) to detect chimera sequences, and then the chimera sequences were removed^87^. Then, we used pick_de_novo_otus.py to pick OTUs by creating an OTU table. Sequences with ≥97% similarity were assigned to the same OTUs. Representative sequence for each OTU was screened for further annotation. For each representative sequence, the Green Gene Database^88^ (http://greengenes.lbl.gov/cgi-bin/nph-index.cgi) was used based on RDP classifier^89^ algorithm to annotate taxonomic information. Observed-species, ACE, Chao1, Shannon, Simpson were calculated with QIIME. ACE and Chao1 were selected to identify community richness. Shannon and Simpson were used to identify community diversity. Principal coordinates analysis (PCoA), Nonmetric Multidimensional Scaling (NMDS) and Unweighted Pair Group Method with Arithmetic mean (UPGMA) clustering were conducted by QIIME software. Linear discriminant analysis effect size (LEfSe) was performed using the online LEfSe program (http://huttenhower. sph.harvard. edu/galaxy/root/index)^90^. The significant difference was calculated using *t*-test.

## Electronic supplementary material

Supplementary figure
