# Supplementary material for: Deciphering the bacterial composition in the rhizosphere of Baphicacanthus cusia (NeeS) Bremek
Source: Sci Rep. 2018 Oct 25;8:15831. doi: 10.1038/s41598-018-34177-1 (PMC6202335; doi:10.1038/s41598-018-34177-1)
Supplement: Supplementary file 1 — Supplementary figure [file 41598_2018_34177_MOESM1_ESM.pdf]

Deciphering the bacterial composition in the rhizosphere of *Baphicacanthus cusia* (NeeS) Bremek

Meijuan Zeng , Yongjia Zhong , Shijie Cai , Yong Diao*

Supplementary figure

Supplementary figure 1. Photograph of *B. cusia*

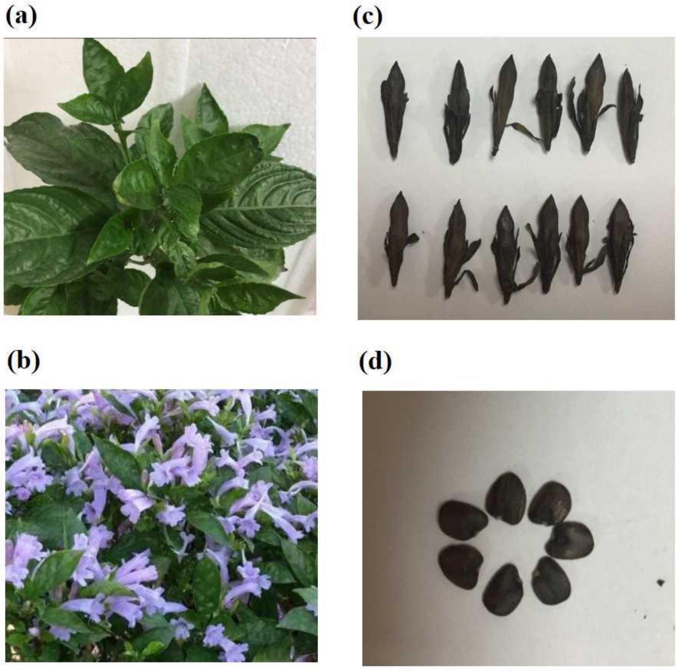

Supplementary figure 1. Photograph of *B. cusia*. (a) Leaves of *B. cusia*. (b) Flowers of *B. cusia.* (c) Pods of *B. cusia*. (d) Seeds of *B. cusia.*
